# Plasma levels of BDNF and EGF are reduced in acute stroke patients

**DOI:** 10.1016/j.heliyon.2022.e09661

**Published:** 2022-06-10

**Authors:** Linda Thøring Øverberg, Elise Fritsch Lugg, Mona Gaarder, Birgitta Langhammer, Bente Thommessen, Ole Morten Rønning, Cecilie Morland

**Affiliations:** a Department of Behavioral Sciences, Faculty of Health Sciences, OsloMet−Oslo Metropolitan University, Oslo, Norway; b Section for Pharmacology and Pharmaceutical Biosciences, Department of Pharmacy, The Faculty of Mathematics and Natural Sciences, University of Oslo, Oslo, Norway; c Department of Physiotherapy, Faculty of Health Sciences, OsloMet−Oslo Metropolitan University, Oslo, Norway; d Research Department, Sunnaas Rehabilitation Hospital, Nesoddtangen, Norway; e Department of Neurology, Division of Medicine, Akershus University Hospital, Lørenskog, Norway; f Institute of Clinical Medicine, University of Oslo, Oslo, Norway

**Keywords:** Biomarkers, Disability, Growth factors, Irisin, Ischemic stroke, Plasticity

## Abstract

Stroke affects almost 14 million people worldwide each year. It is the second leading cause of death and a major cause of acquired disability. The degree of initial impairment in cognitive and motor functions greatly affects the recovery, but idiosyncratic factors also contribute. These are largely unidentified, which contributes to making accurate prediction of recovery challenging. Release of soluble regulators of neurotoxicity, neuroprotection and repair are presumably essential. Here we measured plasma levels of known regulators of neuroprotection and repair in patients with mild acute ischemic stroke and compared them to the plasma levels in healthy age and gender matched controls. We found that the levels of BDNF and EGF were substantially lower in stroke patients than in healthy controls, while the levels of bFGF and irisin did not differ between the groups. The lower levels of growth factors highlight that during the acute phase of stroke, there is a mismatch between the need for neuroprotection and repair, and the brain's ability to induce these processes. Large individual differences in growth factor levels were seen among the stroke patients, but whether these can be used as predictors of long-term prognosis remains to be investigated.

## 1 Introduction

Stroke is the second leading cause of death and a major contributor to acquired disability in the world today [1]. The degree of long-term functional loss after stroke can vary from nearly none to different degrees of impaired motor function, speech loss and reduced cognitive function which may greatly affect the autonomy of the patient. More than 80 million stroke survivors worldwide are currently living with such consequences [2, 3]. Ischemic stroke is caused by an occlusion of a cerebral blood vessel which causes interruption of blood supply to the affected brain area. This rapidly results in tissue necrosis at the stroke core, which may be followed by loss of brain functions, disabilities, or even death. According to the annual report from the World Stroke Organization (WSO), stroke has reached epidemic proportions [2]. Although the incidence and mortality rate of stroke is currently decreasing in high-income countries, the prevalence and the public health burden of stroke in developing countries are expected to rise in the years to come [4, 5]. Given the high mortality rate and probability for long-term disabilities, stroke imposes a considerable economic and social burden on the society, as well as on the stroke patients and their families [6, 7].

Functional outcome after stroke depends on the size of the affected area in the brain and the location, how long the blood supply is reduced or absent for, and whether the stroke is ischemic or hemorrhagic. The degree of initial impairment greatly affects the recovery, but variability between stroke patients with the same lesion type and size sometimes makes an accurate prediction challenging. The individual differences are probably due to a combination of factors, whereof some still are unknown. Hence, the degree of improvement can be hard to predict in the early phase of a stroke.

Brain injury induced by stroke results from complex series of pathophysiological events, including increased release of excitatory transmitters, oxidative stress, inflammation, and cell death. At the same time, cytokines and growth factors that increase the survival of brain cells are released. Growth factors are a group of peptides that stimulates cellular processes, including cell survival, growth, proliferation and differentiation [8]. Growth factors are key regulators of neural plasticity, which is an important part of the repair and regeneration after stroke. Neural plasticity includes processes such as angiogenesis, neurogenesis [9, 10], and rewiring of the brain. All these processes are stimulated by growth factors and are important contributors to the functional recovery after stroke [11, 12, 13]. Irisin, a hormone-like myokine with neurotrophic effects, has also been reported to induce beneficial effects after stroke, including inhibited post-ischemic inflammation, reduced oxidative stress and improved mitochondrial function [14]. In fact, decreased concentrations of irisin are associated with poor functional outcome in ischemic stroke [15].

Growth factors are ubiquitously expressed in the adult brain, and many of them are upregulated in response to ischemia [16]. Irisin is mainly expressed in skeletal muscle but is also produced by brain cells, primarily neurons [17, 18]. Some growth factors remain elevated over several weeks after stroke [19]. Probably the combination of harmful and protective factors released during the acute phase of stroke regulates the balance of neurotoxic and neuroprotective or neuroregenerative processes. The release and effects of growth factors in stroke are subjected to great individual differences and are influenced by a variety of factors including localization of the stroke, genetic factors [20], sex [21] and other−yet unknown−factors. Most growth factors readily pass the blood-brain barrier (BBB), and hence their concentrations in the systemic circulation may be a proxy for the concentration that affects the brain.

Based on the known effects of growth factors and irisin in stroke, treatment strategies to increase regeneration of nerve tissue, for example by supplying larger amounts of growth factors, have received much attention [22]. Although these substances are known to provide positive effects in the healthy brain, the effects of giving them as stroke treatment are less promising. In animal models, post-stroke delivery of brain-derived neurotrophic factor (BDNF) has given promising effects [23]. For various reasons, however, the same effects have not been reached in humans [24]. Similarly, treatments to increase irisin levels are effective in animal models of stroke [14, 25, 26], but have so far not been translated into human stroke patients. The effect of giving basic fibroblast growth factor (bFGF), fibroblast growth factor 2 (FGF-2) and epidermal growth factor (EGF) after stroke is uncertain [27]. Vascular endothelial growth factor A (VEGF A), which regulates the development of blood vessels in the brain, has surprisingly proved to exacerbate the injury of stroke in the acute phase [19], but rescue brain cells in the sub-acute phase [28]. The lack of effect of added growth factors in acute stroke patients may be due to unintentional side effects, low selectivity, low ability to cross the BBB, or difficulty in finding the right dose in transition from animal studies to clinical trials [24]. Intrinsically elevated levels of irisin or growth factors, on the other hand, may be important contributors to−and biomarkers for−recovery after stroke.

**Figure 1:**
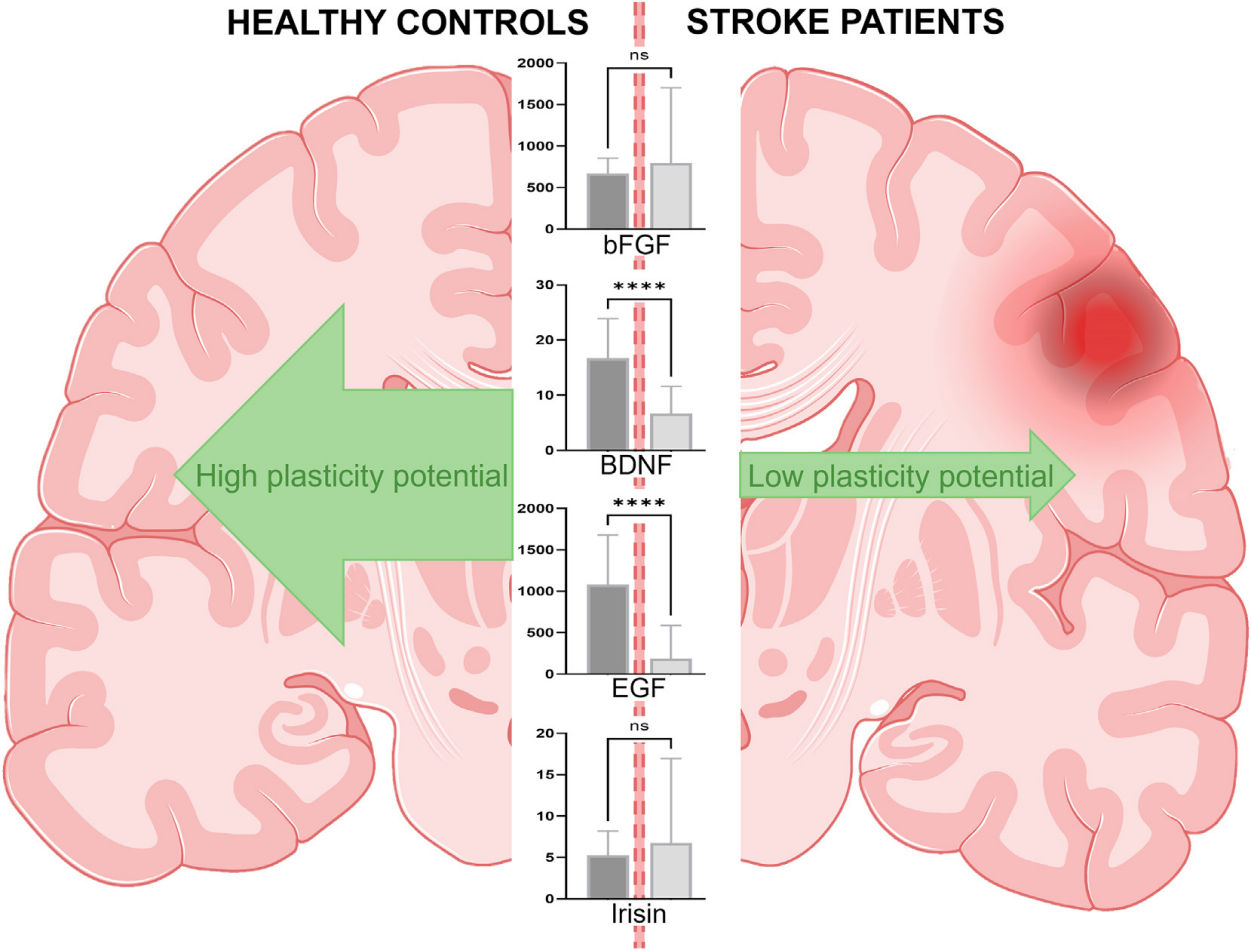
Graphical abstract. Figure partly created with www.BioRender.com.

In the present study we aimed to determine whether differences in plasma levels of irisin and key growth factors (BDNF, bFGF, and EGF) could be detected in acute stroke patients compared to a group of age and gender matched healthy controls (Figure 1).

## 2 Materials and methods

### 2.1 Ethics and approvals

The study was approved by the Regional Committee for Medical and Health Research Ethics, South-Eastern Norway (REC; ID 2018/2555), and the Norwegian center for research data (NSD; ID 539270), and it was registered in the quality assurance system for health and medical research at the University of Oslo, Norway (Helseforsk). The stroke patients were recruited from the health registry and general research biobank for neurological diseases at Akershus University Hospital (Ahus), Norway (REC ID 2011/1015). The study was conducted in accordance with the Declaration of Helsinki of 1964 [29]. The research is reported according to the Biospecimen Reporting for Improved Study Quality (BRISQ) recommendations [30]. The stroke patients were given verbal information about the project and signed a written informed consent the day after admission to the hospital, prior to inclusion and blood sampling. Participation to this study did not affect the treatment of the stroke patients, except for the extra blood sample taken.

All blood samples, case report forms (CRFs), and test scores were de-identified and labeled with a participant number, hence all analyses were performed by researcher(s) who were blinded to the identity of the stroke patients/healthy controls. The paper file with the participant's identity linked to the study ID was kept secured in a safe, separated from the rest of the study data. The CRFs and test scores were kept in a locked drawer in a room with strict access control (card and code to enter the hallway; key to enter the room; separate key to open the drawer).

### 2.2 Study groups and design

**Figure 2:**
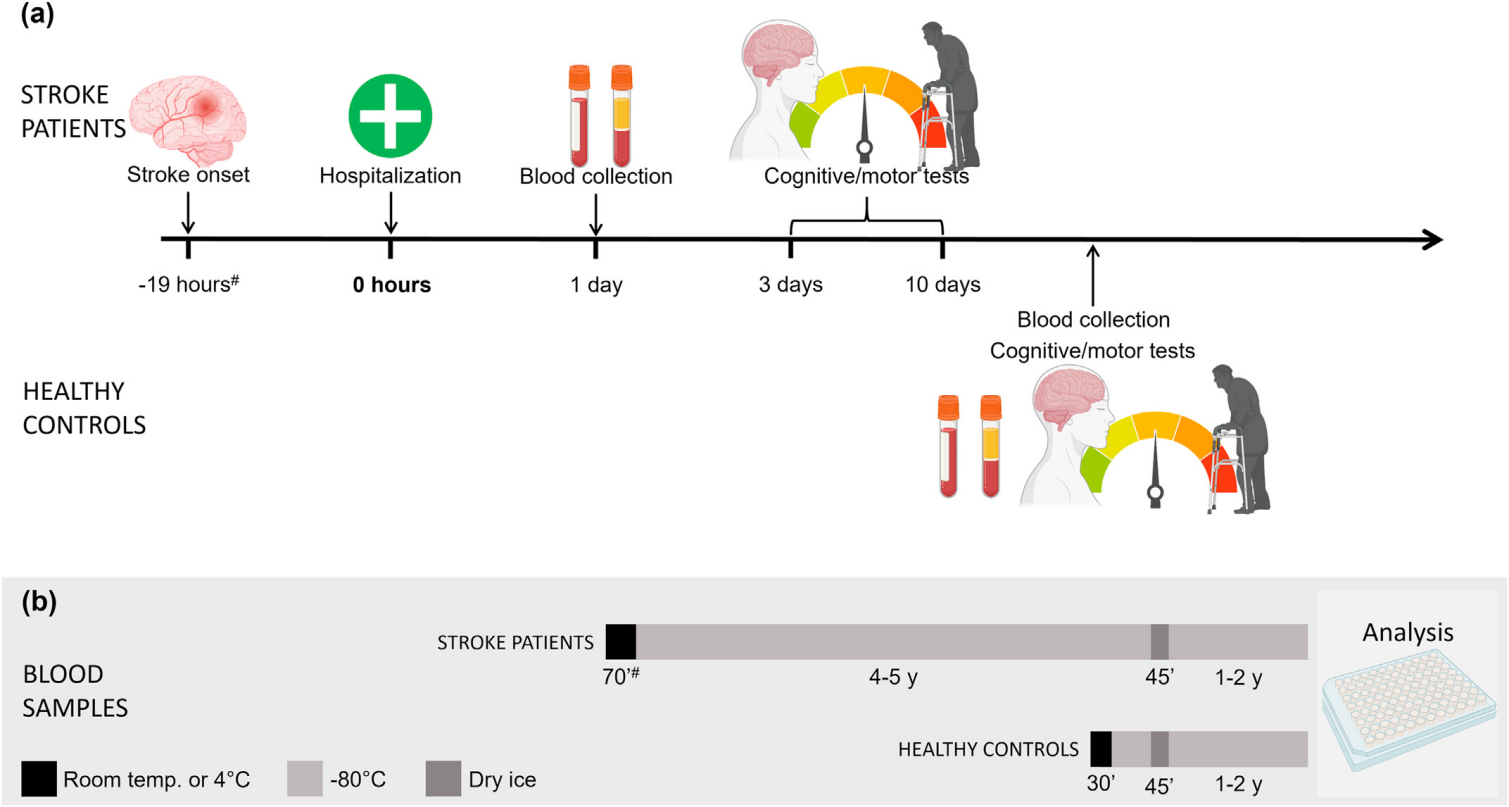
Timeline for the project. (a) Timeline for the stroke patients and the healthy controls. For the stroke patients, the time intervals between stroke onset and hospitalization, and between hospitalization and blood collection and testing are indicated. For the healthy controls, blood sampling and testing were performed on the same day, about 4 years after inclusion of the first stroke patient. (b) Timeline for the plasma samples from stroke patients and healthy controls. The samples were kept at room temperature before centrifugation at +4 °C, and again during pipetting (black). The plasma samples where then kept at -80 °C (grey). During transportation from the biobank to the local lab, the samples were kept on dry ice (dark grey). The samples were then kept at -80 °C (grey) until analysis. #median time; ’minutes. Figure partly created with www.BioRender.com.

The study included 94 subjects, 47 stroke patients and 47 age and gender matched controls (17 males; 30 females in each group). A timeline for the study is given in Figure 2. The stroke patients were selected from the established biobank (REC ID 2011/1015) based on the following inclusion criteria: 1) age >50 years, 2) acute ischemic stroke (ICD-10-CM code: I63) was the main cause for hospitalization, 3) cognitive abilities that allowed patients to give an informed consent to participate, and 4) the time between stroke onset and hospitalization had been noted in the patient journal. The stroke diagnosis was verified by a stroke neurologist based on symptoms and brain imaging, either computer tomography (CT) or magnetic resonance imaging (MRI). Patients with hemorrhagic stroke were not included in the study. The stroke etiology was categorized based on whether the stroke was lacunar, cortical/non-lacunar, or transient ischemic attacks (TIA). Patients with TIA were included in the study only if they exhibited unilateral weakness. The non-TIA strokes were further classified into three size categories: large infarctions (cortical/subcortical infarctions with a volume >10 ml), medium infarctions (cortical/subcortical infarctions with a volume of approximately 5–10 ml), and small infarctions (lacunar or non-lacunar infarctions with a volume <5 ml).

The healthy controls were recruited from fitness centers in the south-eastern Norway. The project leaders gave a verbal presentation of the planned study at the end of fitness classes for elderly people. Other controls were recruited independently of the fitness classes through our network of seniors. Those interested to participate wrote their contact information on a list and were then contacted by e-mail with information about the study. All participants signed a written informed consent on the day of participation, prior to inclusion. The inclusion criteria were age >50 years, healthy (defined by self-reported experience of own health), and cognitive ability that allowed for an informed consent. Exclusion criteria: previous stroke or TIA.

### 2.3 Tests for cognitive and motoric status, and self-sufficiency

Cognitive abilities were tested with mini-mental state examination (MMSE), trail making test A (TMTA) and trail making test B (TMTB). MMSE includes tests of orientation, attention, memory, language, and visual-spatial skills; TMTA and TMTB measure executive functions such as visual attention and psychomotor tempo [31, 32]. For the stroke patients, these tests were performed between day three after admission and the time they were discharged from the hospital (the patients were normally hospitalized for 4–10 days). For the healthy controls, these tests were performed directly prior to the blood sampling. MMSE was performed as described by Folstein and colleagues [33] with the Norwegian revisions by Strobel and Engedal [34]. In the TMTA, the time (in seconds) used by each person to connect 25 numbered circles in an ascending order with a continuous line was measured. In TMTB, the 25 circles were labeled with either a number or a letter, and the time (in seconds) to connect the circles, with a continuous line, alternating between numbers and letters and taking both series in ascending order, from 1-A-2-B etc., was measured. In addition, before discharge from the hospital, the degree of disability was measured according to the Barthel activities of daily living (ADL) Index (0–20) [35, 36] and the mRS [37].

### 2.4 Blood collections and plasma analyses

The sample of venous blood was drawn from the stroke patients between noon and 1 pm on the day after admission to the hospital. The median time between symptom onset and hospitalization was 19 h but varied from 1 h to >240 h (Figure 2; Table 1). The blood samples were collected in de-identified (labeled with the participant number only) BD vacutainer tubes containing K_2_ dipotassium ethylenediaminetetraacetic acid (K_2_EDTA) 10.8 mg, ref. 367864). The tubes were gently inverted 10 times and centrifuged within 10–15 min (5810 R, serial number 0036330, Eppendorf Nordic, Hoersholm, Denmark) at 1000 g for 5 min, with temperature of +4 °C. The supernatant (plasma) was aliquoted within 45–140 min (median: 70 min), stored at -80 °C in the biobank for four to five years, and were kept on dry ice for 30 min during shipment before further analysis. From the healthy controls, 6 mL of venous blood was collected from the antecubital area of the arm after at least 30 min of rest. The blood was collected in BD vacutainer tubes and the plasma preparation was performed as described above. The plasma was gently pipetted in 200 μL aliquots in pcr tubes (VWR, kat.nr: 732-0676) and stored at -80 °C until further analysis.

**Table 1:** Summary characteristics for the healthy controls (Control) and stroke patients (Stroke).

Description	Control	Stroke
number of participants (n)	47	47
age, mean ± SD	70.5 ± 7.0	70.3 ± 7.8
females, n (%)	30 (64%)	30 (64%)
stroke type		
lacunar, n (%)		25 (53%)
cortical/non-lacunar, n (%)		15 (32%)
TIA, n (%)		7 (15%)
stroke size		
small, n (%)		32 (80%)
medium, n (%)		4 (10%)
large, n (%)		4 (10%)
Time: stroke onset to hospitalization		
median (hours)		19
min-max (hours)		1- >240

The plasma concentration of BDNF, bFGF, EGF, and irisin were analyzed by sandwich enzyme-linked immunosorbent assay (ELISA) (R&D Systems Inc. Minneapolis, USA). The optimal dilution of the plasma samples for each analysis was identified in a separate test where four different dilutions of plasma were tested. The following ELISA kits were used, and plasma was diluted as indicated: Human BDNF (DY248), dilution: 1:40; human EGF (DY236-05), dilution: 1:5 for the stroke patients; 1:20 for the healthy controls; human FGF (DY233), dilution: 1:10; human irisin (DY9420-05), dilution: 1:5. The analyses were conducted according to the instructions of the manufacturer. All plasma samples were assayed in duplicates, and samples from stroke patients and healthy controls were evenly distributed on each plate. An intra-assay coefficient of variation (CV) below 20% between the duplicates was considered acceptable, but in most cases, it was well below 10%. Samples where the CV exceeded 20% were reanalyzed. The blood samples were subjected to a maximum of three freeze-thaw cycles, and the time of each thaw was about 1 h (on ice).

### 2.5 Statistical analysis

Statistical analyses were performed using GraphPad Prism 9 (GraphPad Software Inc., California, USA). One healthy control was excluded from the analysis as this person had plasma levels several folds above the rest of the healthy controls cross all analyses. To keep the age and sex distribution of both groups equal, the matched stroke patient was also excluded. Some individuals were identified as statistical outliers for single growth factor/irisin analysis. As individual differences are presumably important for stroke recovery, these individuals were included in the analyses and graphs. A separate analysis was conducted to verify that inclusion of these individuals did not affect the conclusion of the statistical comparisons. The normality of each dataset was tested using the Shapiro-Wilk test, and nonparametric tests were found to be applicable. Comparisons between the stroke patients and the healthy controls were analyzed by the Mann-Whitney U test. When comparing more than two groups, a Kruskal-Wallis test was performed. The significance level was set to <0.05.

## 3 Results

The study group consisted of 47 stroke patients and 47 age and gender matched subjects in the control group. The participants ranged from 53-83 years of age; the majority (85%) were between 60 and 80 years of age (Table 1).

The stroke patients on average had a modified Rankin Scale (mRS) score consistent with slight disability [37] (Table 2). The mean Barthel ADL score reflected a low need for help, but seven of the 47 stroke patients had a Barthel ADL score of 10–19 (reflecting a moderate need for help), and six scored below 9 (reflecting a high need for help) [35] (Table 2). The MMSE score of both the stroke patients (26 ± 4.5 of 30; mean ± SD) and the healthy controls (29 ± 1.0 of 30) were considered normal [33], but the score was significantly lower among the stroke patients than the healthy controls (p < 0.0001; Mann-Whitney U test; GraphPad Prism). Among the 39 stroke patients who were tested by MMSE, 27 had a score of 25–30, suggesting no cognitive impairment; 8 had a score of 21–24, consistent with mild cognitive impairment, and 4 patients scored 10–20, consistent with moderate cognitive impairment. None of the patients scored below 10 (severe cognitive impairment). In the healthy controls, all participants scored 27 or above (Table 2).

**Table 2:** Results from cognitive, motoric, and self-sufficiency tests of the healthy controls (Control) and the stroke patients (Stroke).

Description	Control	Stroke
mRS^1^	-	
mean ± SD		1.9 ± 1.4
median (n)		2 (47)
MMSE^2^		
mean ± SD	29.0 ± 0.97	26.1 ± 4.5
median (n)	29 (47)	27 (39)
TMTA		
mean (sec) ± SD	37.6 ± 14.3	60.0 ± 35.1
Median (n)	33.3 (47)	46.5 (26)
TMTB		
mean (sec) ± SD	89.7 ± 47.4	131 ± 64.5
median (n)	75 (47)	114 (25)
Barthel ADL score^3^	-	
mean ± SD		17.5 ± 4.9
median (n)		20 (47)

1 mRS: 0–5 scale; 0 = no symptoms; 5 = severe disability; constant care needed (6 = dead).

2 MMSE: maximum score: 30.

3 Barthel ADL score: maximum score: 20 (= self-sufficient).

Time spent to complete TMTA [32, 38] (60.0 ± 35.2 s) for the stroke patients was longer than for the healthy controls (37.6 ± 14.3 s; p = 0.0006; Mann-Whitney U test; GraphPad Prism). The time to complete the TMTB [32] varied largely between the individuals but was longer for stroke patients (131 ± 64.5 s; mean ± SD) than for the healthy controls (89.7 ± 47.4 s; mean ± SD; p = 0.0015; Mann-Whitney U test; GraphPad Prism) (Table 2).

### 3.1 BDNF levels were lower in stroke patients than in healthy controls

**Figure 3:**
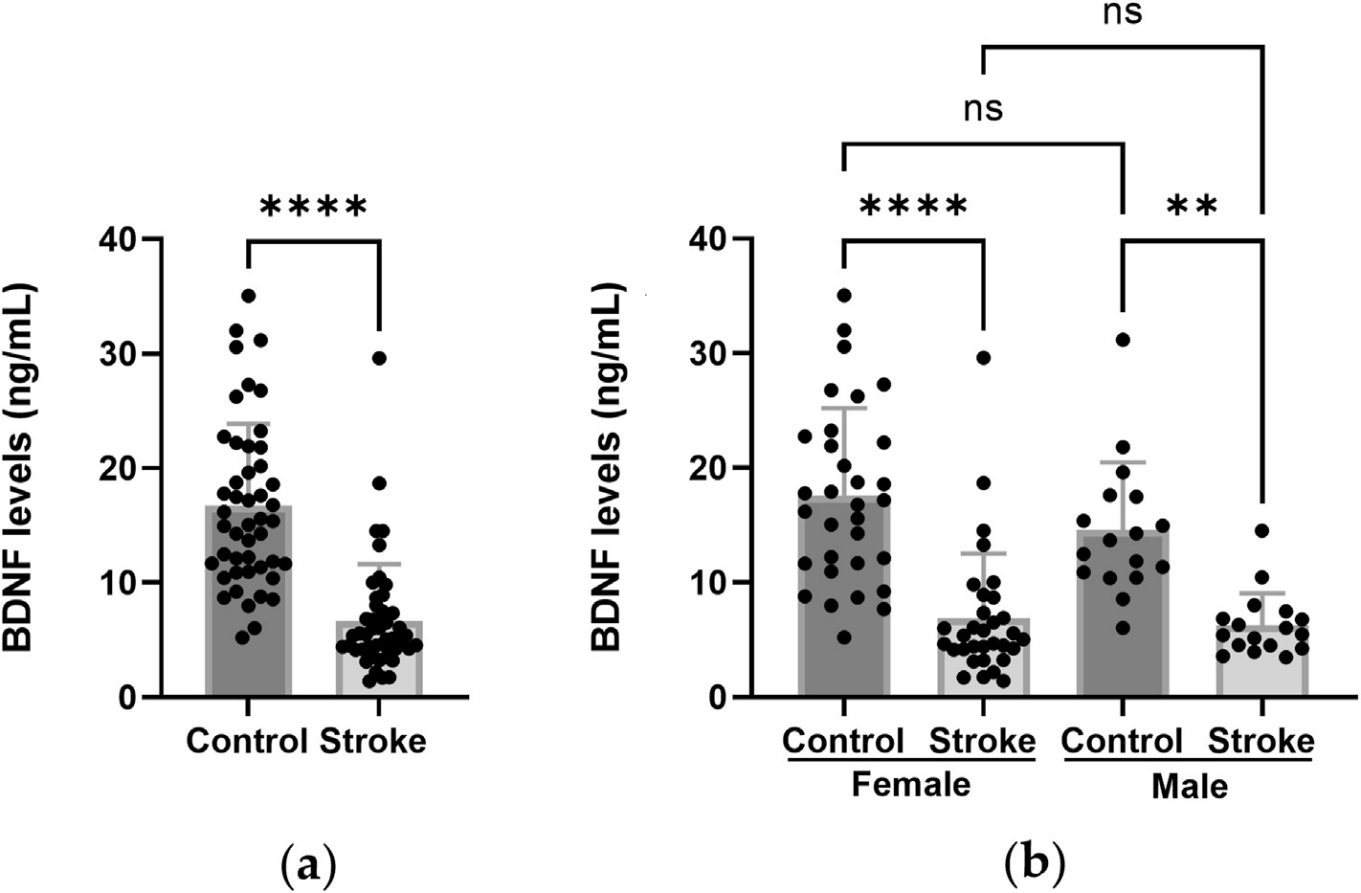
Plasma levels of BDNF is reduced in stroke patients. (a) Plasma levels of BDNF (ng/mL) in healthy controls and stroke patients; (b) Plasma levels of BDNF (ng/mL) in healthy male and female controls and stroke patients. ∗∗p < 0.01; ∗∗∗∗p < 0.0001; ns: not statistically significant (in (a): Mann-Whitney U test, in (b): Kruskal-Wallis test; GraphPad Prism).

In the stroke patients, the levels of BDNF ranged from 1.43 to 29.61 ng/mL, and in the healthy controls, the range was 5.23–35.06 ng/mL. The plasma BDNF levels in the stroke patients (6.69 ± 4.92 ng/mL; mean ± SD) were significantly lower than in the age and gender matched control group (16.70 ± 7.20 ng/mL; mean ± SD; p < 0,0001; Mann-Whitney U test, GraphPad Prism) (Figure 3a).

A sub-analysis was performed to establish whether males and females differed in the BDNF levels. The data were segregated based on sex (n = 30 females and 17 males in each group) and reanalyzed (Figure 3b). There was no difference between males and females in any of the groups (p > 0.99 for both comparisons). In females, the BDNF levels were 61% lower in the stroke patients than in the healthy controls, and in males the BDNF levels were 57% lower in the stroke patients than in the healthy controls. Females: 6.88 ± 5.63 ng/mL (mean ± SD) in stroke patients versus 17.58 ± 7.64 ng/mL in healthy controls (p= <0.0001; Kruskal-Wallis test, GraphPad Prism). Males: 6.29 ± 2.78 ng/mL in stroke patients versus 14.59 ± 5.88 ng/mL in healthy controls (p = 0.0013; Kruskal-Wallis test, GraphPad Prism).

### 3.2 BDNF levels were largely unaffected by age

**Figure 4:**
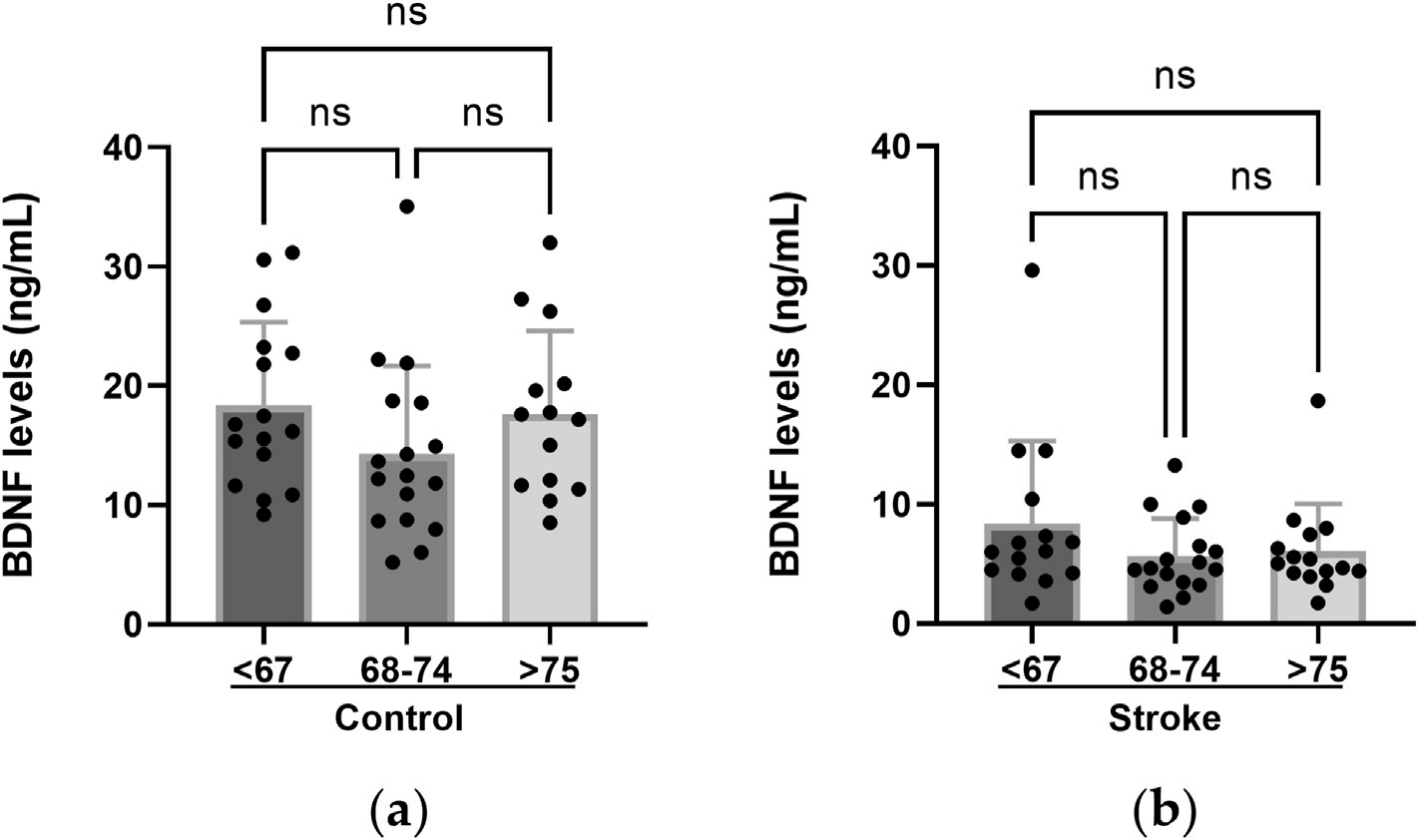
Plasma levels of BDNF are unaffected by age. Plasma levels of BDNF (ng/mL) in different age groups of (a) healthy controls; (b) stroke patients. ns: not statistically significant (Kruskal-Wallis test; GraphPad Prism).

To study whether the BDNF levels were affected by age, the subjects were categorized into three age groups: <67 years (n = 15; 16 stroke patients and healthy controls, respectively), 68–74 years, (n = 17; 17); >75 years (n = 15; 14). The BDNF levels did not change in relation to age, neither in the stroke patients nor the healthy controls (Figure 4). The levels of BDNF were lower in the stroke patients than in the healthy controls, irrespectively of age. The difference slightly increased with age: In the youngest age group (<67 years of age) BDNF levels in stroke patients were 45.6% of the levels measured in the healthy controls. In patients who were 68–74 years of age, this percentage was 39.6%, and in patients >75 years of age it was 34.7%.

### 3.3 BDNF levels were unaffected by stroke size

**Figure 5:**
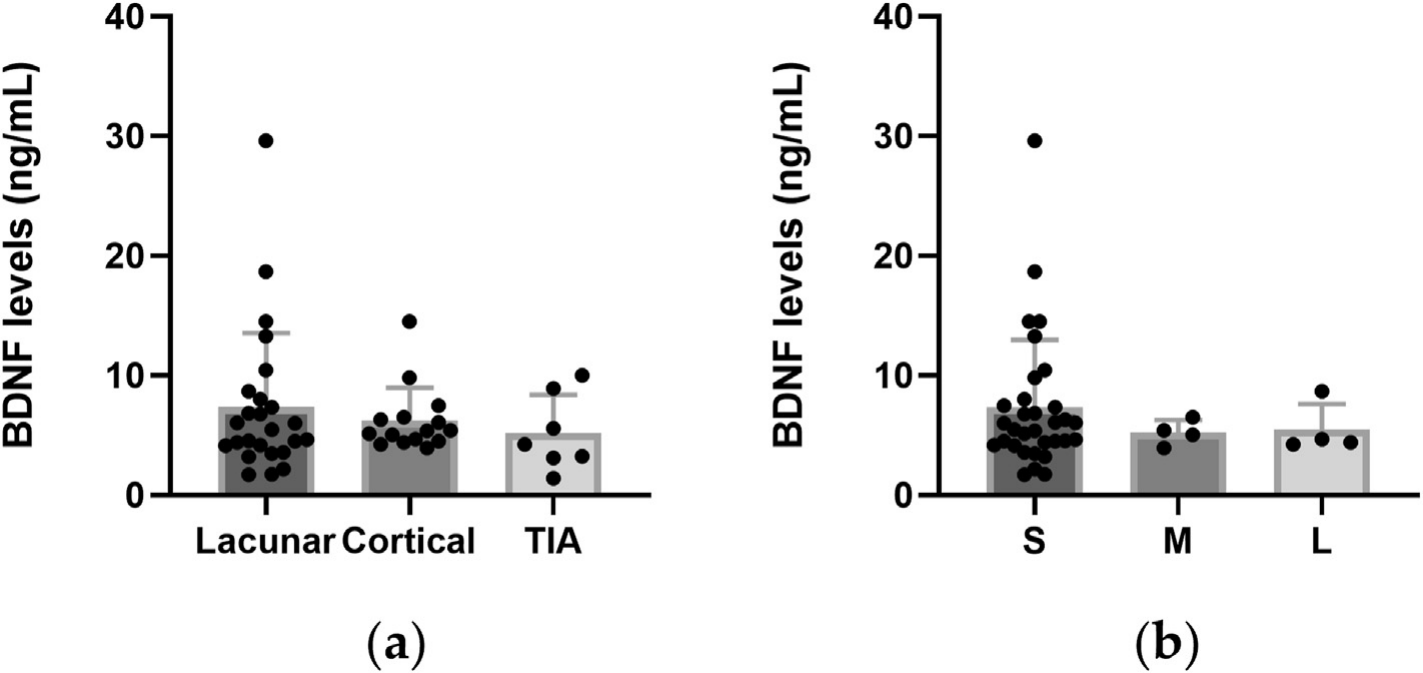
Plasma levels of BDNF are unaffected by stroke type. Plasma levels of BDNF (ng/mL) segregated by (a) stroke type; (b) stroke size (p = 0.57 and p = 0.81, respectively; Kruskal-Wallis test; GraphPad Prism).

The stroke patients (n = 47) were classified based on whether the lesion was lacunar, cortical/non-lacunar or TIA (Table 1). The levels of BDNF did not differ between the different stroke types (Figure 5a; p = 0.57, Kruskal-Wallis test, GraphPad Prism). The non-TIA stroke patients (n = 40) were further divided based on the lesion size into small (n = 32), medium (n = 4), large (n = 4). BDNF levels were not dependent on the stroke size (Figure 5b; p = 0.81, Kruskal-Wallis test, GraphPad Prism).

### 3.4 bFGF levels were marginally higher in stroke patients than in healthy controls

**Figure 6:**
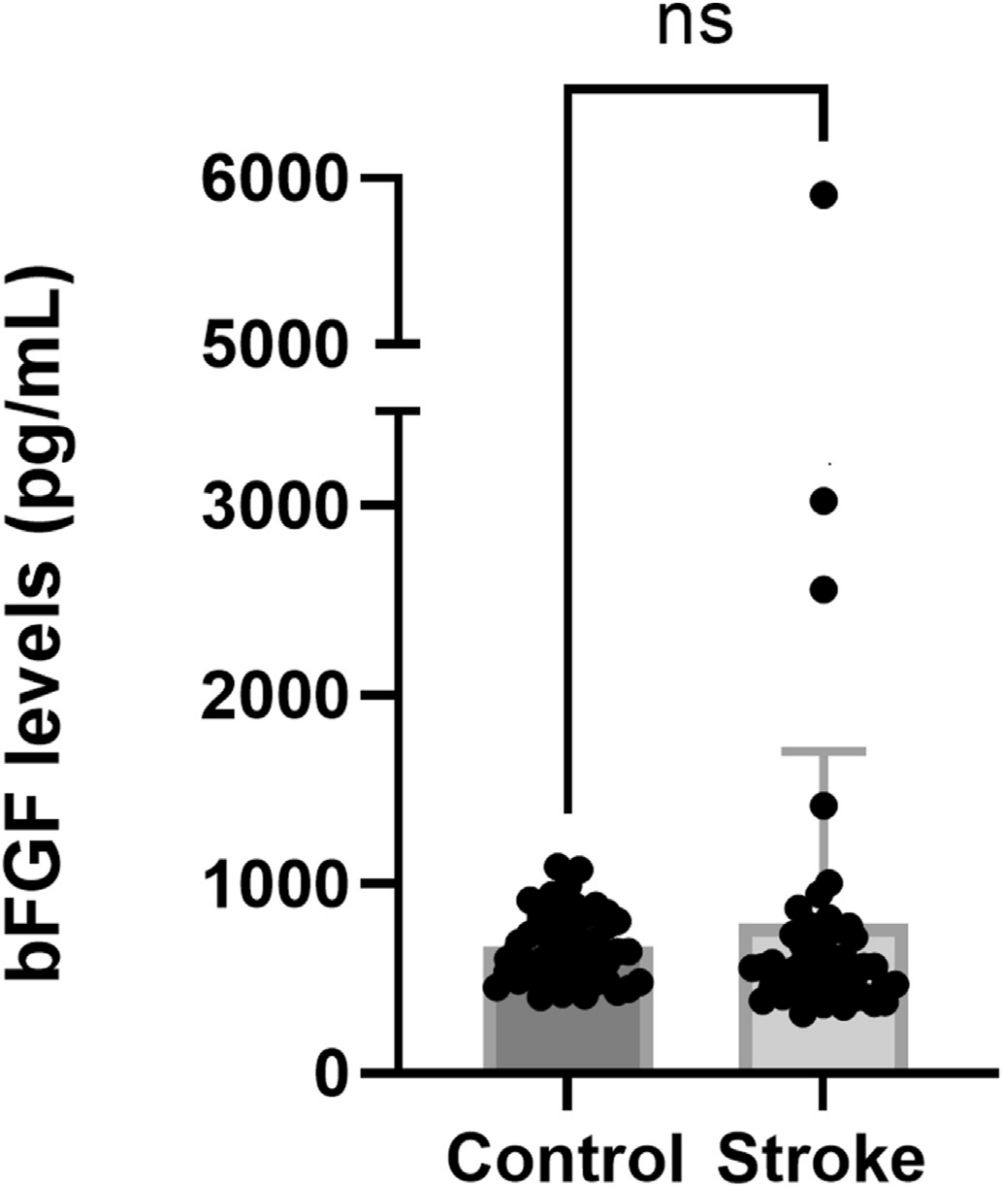
Plasma levels of bFGF. Plasma levels of bFGF (pg/mL) in healthy controls and stroke patients. ns: not statistically significant (Mann-Whitney U test, GraphPad Prism).

The bFGF levels were marginally higher in the stroke patients (795 ± 908 pg/mL; mean ± SD) than in the healthy controls (671 ± 184 pg/mL; p = 0.068; Mann-Whitney U test, GraphPad Prism; Figure 6). Four stroke patients were identified as statistical outliers, but these were included in the analysis. A separate analysis confirmed that the inclusion of these patients did not change the conclusion. The bFGF levels did not differ between males and females, neither for the stroke patients nor for the healthy controls (p > 0.95; Kruskal-Wallis test; GraphPad Prism; data not shown). Furthermore, the bFGF levels were not affected by the stroke size (p > 0.65; Kruskal-Wallis test; GraphPad Prism; data not shown).

### 3.5 EGF levels were lower in stroke patients than in healthy controls

**Figure 7:**
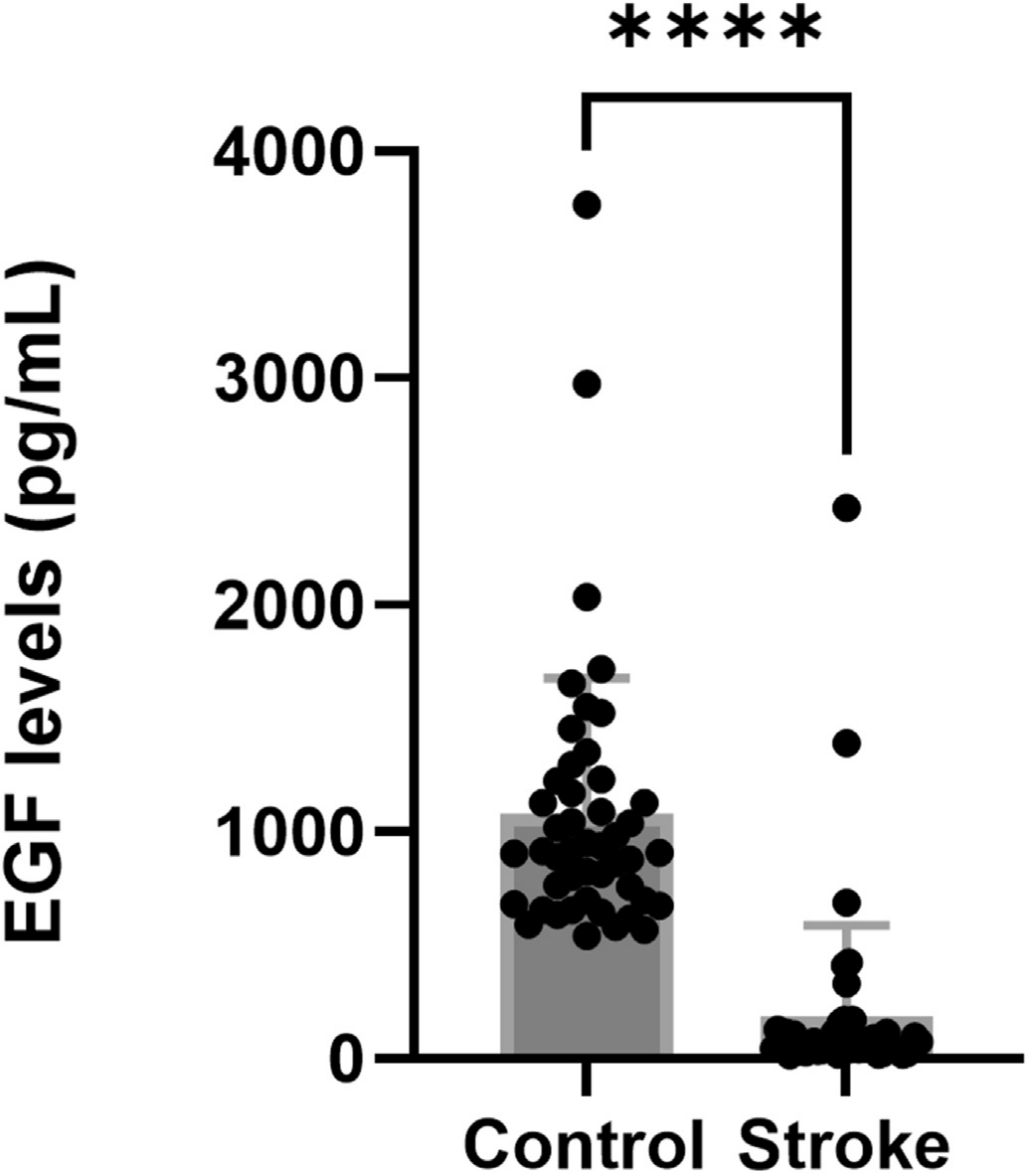
Plasma levels of EGF. Plasma levels of EGF (pg/mL) in healthy controls and stroke patients. ∗∗∗∗: p < 0.0001; (Mann-Whitney U test, GraphPad Prism).

The EGF levels varied largely between the participants. In stroke patients, plasma EGF varied between 16.5 and 2428 pg/mL, a 15-fold difference. In the healthy controls the EGF levels varied almost 7-fold, ranging from 541 to 3766 pg/mL. The level of EGF was almost 6-fold lower in the stroke patients (184 ± 403 pg/mL (mean ± SD) than in the healthy controls (1080 ± 599 pg/mL; p < 0.0001; Mann-Whitney U test; GraphPad Prism) (Figure 7). Some individuals (stroke: n = 6; control: n = 2) were identified as statistical outliers and had EGF levels several folds higher than the mean levels in the respective groups. These were included in the analysis, but a separate analysis was performed to confirm that the inclusion of these individuals did not change the conclusion.

As reported for BDNF and bFGF, the levels of EGF did not differ between males and females, neither in the healthy controls nor among the stroke patients (p > 0.99; Kruskal-Wallis test; GraphPad Prism; data not shown). Furthermore, the EGF levels were unaffected by the stroke size (p = 0.67; Kruskal-Wallis test; GraphPad Prism; data not shown).

### 3.6 Irisin levels were similar in stroke patients and healthy controls

**Figure 8:**
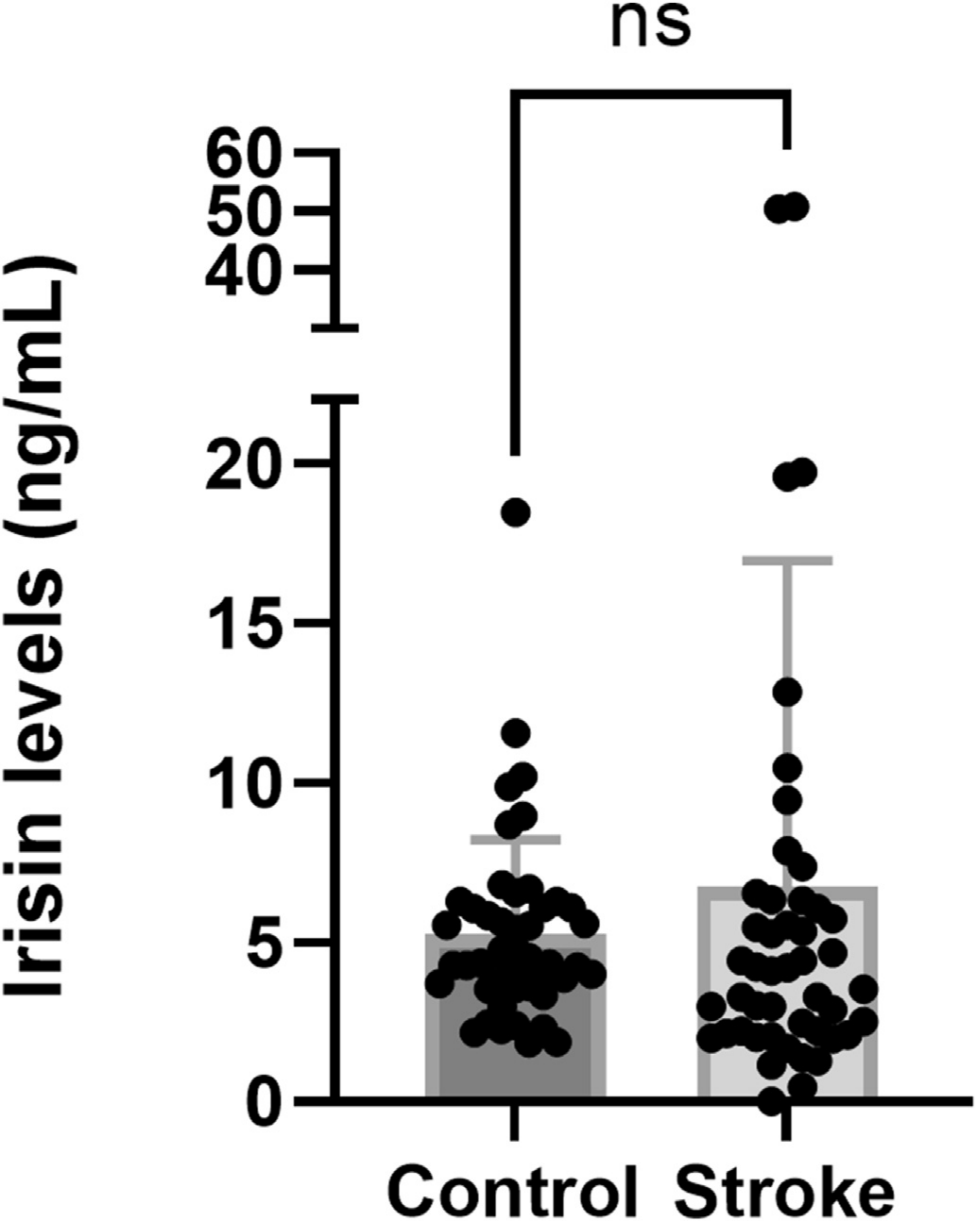
Plasma levels of irisin. Plasma levels of irisin (ng/mL) in healthy controls and stroke patients. ns: not statistically significant (Mann-Whitney U test, GraphPad Prism).

The irisin levels did not differ between the stroke patients (6.74 ± 10.22 ng/mL) and the healthy controls (5.27 ± 2.94 ng/mL; mean ± SD; p = 0.14; Mann-Whitney U test; GraphPad Prism) (Figure 8). The irisin levels, however, varied greatly, especially among the stroke patients (ranging from undetectable levels to 50.90 ng/mL). The corresponding range in the healthy controls was 1.85–18.47 ng/mL. The variation in irisin levels in stroke patients was not related to differences in stroke size (p = 0.23; Kruskal-Wallis test; data not shown) and was similar in males and females (Kruskal-Wallis test; p = 0.50; GraphPad Prism; data not shown).

## 4 Discussion

The present study demonstrates that plasma levels of the growth factors BDNF and EGF were 2.5- and 5.7-fold lower, respectively, in acute stroke patients than in age and gender matched healthy controls. The levels of bFGF and irisin did not differ between the groups. The levels of growth factors and irisin was unaffected by sex and age. Somewhat surprisingly, we did not find any correlation between the levels of growth factors or irisin and the type of stroke (lacunar, cortical/non-lacunar or TIA) or the lesion size. The low numbers of medium and large infarct sizes, however, calls for caution when drawing a conclusion of no association.

The stroke patients participating in the present study all had cognitive abilities to give an informed consent. The most severe stroke cases, including those who were not capable of approving participation in the study, were excluded. In the cognitive, motoric and self-sufficiency tests, the stroke patients were out-performed by the healthy controls as expected.

Although, stroke is known to be a major contributor to both physical and cognitive decline, we cannot conclude with certainty that the differences in cognitive abilities between the stroke patients and the healthy controls were caused by the stroke *per se*; the possibility that the stroke patients had a lower cognitive score even prior to the stroke must be taken into consideration. In fact, published data indicate that 10% of stroke patients have pre-stroke dementia [39].

The lower levels of BDNF and EGF found in the stroke patients compared to the healthy controls cannot with certainty be ascribed to stroke *per se*; the stroke patients could in theory have lower levels of these growth factors independently of the stroke. The differences are, however, quite large, making it more plausible that they are caused by a major event like stroke than by natural variation. We detected a substantial decrease in plasma BDNF in the stroke patients compared to the healthy controls. Only a few studies have analyzed plasma levels of BDNF after stroke. One reason for this may be that K_2_EDTA, which is commonly used as an anticoagulant in the plasma samples−including in the present study−may activate platelets. This can result in the release of BDNF from these platelets, which in turn may mask differences in BDNF released from the brain in response to stroke.

Our findings are, nevertheless, in line with a recent meta-analysis [40] summarizing seven publications where BDNF serum levels in stroke patients during the acute phase were compared to control groups. The magnitude of difference in BDNF levels between stroke patients and healthy controls vary from study to study: Algin and colleagues found that BDNF serum levels in stroke patients at the time of admission to the hospital, were 3.8-fold lower than in a control group who were admitted to the emergency department for non-neurological reasons [41]. Similarly, Chaturvedi and colleagues reported a 2-fold lower serum BDNF in stroke patients compared to in healthy controls [42]. In both studies, the blood was collected at admission, whereas in the present study, blood samples were drawn the day after admission. In another study, the levels of BDNF in stroke patients measured within 10 days after stroke onset (median: 4 days) were found to be reduced compared to the controls [43]. This illustrates the robustness of the BDNF decrease after stroke. Nevertheless, some studies did not find reduced levels of BDNF in stroke patients the first day [44], or seven days after stroke [45]. In fact, even increased BDNF levels have been reported in stroke patients compared to controls [46]. The former study did, however, report that plasma BDNF levels at day one were significantly lower in patients who ended up with a Barthel ADL index score ≤80 on day five after stroke onset than in those with a Barthel ADL index score >80 [44]. In the present study a Barthel ADL scale from 0-20 was used, but these scores can easily be converted to the 1–100 index scale: The average Barthel ADL score in our study was 87.5, and 10 patients had a score >80. The stroke patients participating in the present study were relatively homogenous regarding the Barthel ADL scores which may explain why we did not see a correlation between BDNF levels and Barthel ADL scores. The present study is, however, in line with the majority of studies showing reduced BDNF levels in stroke patients compared to healthy controls.

The robust reduction in BDNF levels found in most studies where acute ischemic stroke patients are compared to controls, combined with the known neuroprotective/neurodegenerative effects of BDNF, suggest that means to increase BDNF levels may be effective therapeutic targets in stroke. Administration of BDNF per se, however, is precluded by a low bioavailability of BDNF in the brain [44]. This reflects a combination of low ability to cross the BBB along with a short distribution time in brain tissue. Means to increase endogenous brain-intrinsic BDNF levels may prove to represent more promising therapeutic approaches. So far no BDNF-increasing therapy has reached clinical use in stroke patients.

In the present study there was no correlation between BDNF levels and stroke size or between BDNF levels and Barthel ADL scores in stroke patients. It should be noted that the sample size, especially for the medium and large stroke sizes, is limited and hence these data are encumbered by uncertainty. Furthermore, in the present study, the stroke size was measured retrospectively from MR images obtained 4–5 days after stroke onset. Despite the fact that BDNF levels decrease with age in the normal brain [45, 47, 48], no correlation between age and BDNF levels were found in the present study. Again, the sample size in each age group were limited, bringing uncertainty to the conclusion. Furthermore, we did not detect different BDNF levels in males and females. This is supported by findings in humans [49] and rodents [50], even though it is known that estrogen increases BDNF expression while testosterone decreases it [51]. Taken together, publications report lower BDNF levels in plasma or serum from stroke patients compared to controls during the early phase of a stroke. Supporting a role of BDNF in early stroke recovery, the val66met single nucleotide polymorphisms (SNP) of the *bdnf* gene is among the top polymorphisms implicated in stroke risk and prognosis [52].

In the present study, bFGF levels were marginally higher in the stroke patients than in the healthy controls (p = 0.068). Other studies have shown increased serum bFGF levels when comparing stroke patients to a control group: Guo and colleagues found elevated bFGF levels in serum samples obtained within 48 h after ischemic stroke; the bFGF levels peaked at day three and reminded elevated for 14 days [53]. Elevated levels of bFGF in stroke patients at day three after stroke was confirmed by Golab-Janowska and colleagues even when traditional vascular risk factors were controlled for [54]. Elevated bFGF levels in plasma or serum is consistent with a reported upregulation of bFGF in the brain in response to experimental ischemia in rodents [55, 56] and postmortem in the brain of patients who died 24 h to 43 days after acute ischemic stroke [57]. In the permanent medial cerebral artery occlusion (pMCAO) model in rats, bFGF treatment promote neuroprotection and neurogenesis: Intravenous bFGF injections at 2 h after pMCAO induction resulted in improved functional outcome (rotarod performance) and a substantial reduction of the infarct volume [58]. Intracisternal administration of bFGF at 24 and 48 h after pMCAO led to an increased number of BrdU positive cells in the subgranular zone but did not cause reduced stroke volume [59]. Serum bFGF levels have previously been reported to correlate positively with the infarction size [53]. Such a correlation could not be detected in the present study, perhaps due to the relatively low number of patients with medium or large stroke sizes. A positive correlation between the peak bFGF level and improvement in neurological function between day two and day 20 after stroke has been reported [53], indicating that bFGF may prove to be an early biomarker for progression after stroke.

Through activation of the EGF receptor (EGFR), EGF regulates proliferation and DNA repair. To the best of our knowledge, circulating EGF levels have not previously been investigated in the acute phase of stroke in humans, and effects of EGF in stroke therapy is also missing. In a rat model of stroke, however, intraventricular infusions of EGF and erythropoietin (EPO) together promoted regeneration of the injured neocortex and reversed motor function deficits [60]. Neither EGF nor EPO was able to induce this affect alone. Furthermore, EGFR levels are reported to increase in the penumbra surrounding the stroke core in rodents [61]. In the present study we found that EGF levels in plasma from the stroke patients were nearly 6-fold lower than the levels in the age and gender matched control group. This likely contributes to a reduced capacity for plasticity and tissue regeneration, suggesting that therapies to increase EGF levels during the acute phase of stroke would be beneficial. In order to conclude whether EGF represent a therapeutic target in stroke, the data from the current study need to be confirmed in additional studies, perhaps also including persons with more severe strokes. As described, the evidence for neuroprotective effects of EGF in stroke derives from animals and cell cultures, and the translational value to human patients needs to be determined. We did not detect age or sex dependent changes in EGF levels in the present study, and EGF levels did not depend on the stroke size. The latter may reflect that most of our stroke patients had small lesion sizes, and the limited number of patients with medium or large strokes makes it challenging to detect statistical correlation between EGF levels and stroke size. We therefore cannot conclude whether such a correlation exists, or if the difference in EGF levels between stroke patients and healthy controls would have been even larger if patients with more severe strokes had been included.

Irisin is a myokine released by skeletal muscle during exercise and is known to induce the transformation of white adipocytes to brown adipocytes [62]. During the 10 years since it's discovery, irisin has been implicated in neuroprotection in several neurological diseases, including stroke [63]. Plasma irisin levels have been shown to correlate with levels of irisin in the cerebrospinal fluid (CSF) of healthy humans [64], highlighting the relevance of measuring plasma irisin in stroke patients.

Most studies so far have focused on differences of irisin between groups of stroke patients, and to the best of our knowledge, only one study has reported serum irisin levels in stroke patients compared to a control group of approximately the same age [65]. In their study, Kazimierczak-Kabzińska and colleagues detected significantly lower levels of irisin in stroke patients compared to the control group. However, their control group contained a higher fraction of males, who on average were younger than the stroke patients. Whether or how this bias affects the result is hard to interpret as age has been reported to be negatively correlated with plasma and CSF irisin levels, and irisin have been reported to be higher in males than in females [64]. Further complicating the matter, body mass index−a major risk factor for stroke−is negatively correlated with CSF levels of irisin. Consistent with the results of the present study, Kazimierczak-Kabzińska and colleagues also did not detect differences in irisin levels based on age or sex. This lack of detected correlation may reflect the relatively low number of participants in both studies. Two studies from Chinese populations reported negative correlations between levels of irisin and neurological outcome (measured by the National Institutes of Health Stroke Scale (NIHSS) at admission) and between irisin and stroke volume [66]. Furthermore, serum levels of irisin measured the morning after hospitalization were higher in patients who presented high functional outcomes (measured by mRS) at three months after stroke [66], or six months after stroke [67]. Higher serum irisin levels at the time of admission were also positively correlated to survival for three months [66] or six months [67] post-stroke.

In the present study irisin levels did not differ between the healthy controls and the stroke patients. This finding was somewhat surprising based on the publications presented above. In addition, irisin has been suggested to stimulate the expression of BDNF [18], but in the present study BDNF levels were decreased in stroke patients irrespectively of the unaltered irisin levels. The irisin levels, however, varied between individuals of the same group about 10-fold in the healthy controls, and from undetectable levels to 50.9 ng/mL in the stroke patients. Based on the correlations of plasma irisin levels with long-term functional and neurological outcomes, the large variation in plasma irisin levels may be of interest in itself, as irisin levels may prove to be a suitable biomarker for long-term stroke outcome. In addition, irisin levels did not differ neither between males and females, nor between different age groups, types of stroke, or stroke sizes. The latter finding is supported by Wu and co-workers [66], who also did not detect correlations between age or sex of the patients, or stroke subtype distribution with irisin levels. In healthy humans, however, plasma levels of irisin were observed to increase with age and to be higher in males than in females [64].

We cannot conclude with certainty that irisin levels were unaffected by the stroke, as differences in the levels between the two groups could in theory have been present before stroke onset, hence masking any changes in the stroke patients in response to the stroke. Low CSF or plasma irisin levels have been associated with risk factors for stroke, like high body mass index, high cholesterol levels or diabetes mellitus type II [67]. These risk factors are expected to be higher among the stroke patients than the healthy controls, so if a baseline difference in irisin levels was present, irisin would likely be lower in stroke patients. Based on the correlation between low irisin levels and stroke severity, an increase in irisin in response to stroke (which would be necessary to compensate for lower baseline irisin) is not likely. Hence, we conclude that in the present study irisin levels did not differ between stroke patients and healthy controls.

## 5 Conclusion

In the present study we measured plasma levels of known regulators of neuroprotection and repair in acute ischemic stroke patients and compared them to the plasma levels in healthy age- and gender matched controls. We found that the levels of BDNF and EGF were lower in the stroke patients than in the healthy controls, while the levels of bFGF and irisin did not differ. Furthermore, we found large individual differences in irisin and growth factor levels, which may reflect idiosyncratic mechanisms affecting post-stroke recovery. Taken together, the data suggest that during the acute phase of stroke, there is a mismatch between the need for neuroprotection and repair, and the brain's ability to induce these processes. Most of the patients in the current study had mild strokes. Further studies with larger sample sizes should be performed to confirm the current findings -including their applicability for more severe stroke cases- and demonstrate whether variations in these growth factor levels may be a predictor for post-stroke outcome.

## Declarations

### Author contribution statement

Linda Thøring Øverberg: Conceived and designed the experiments; Performed the experiments; Analyzed and interpreted the data; Wrote the paper.

Cecilie Morland: Conceived and designed the experiments; Analyzed and interpreted the data; Contributed reagents, materials, analysis tools or data; Wrote the paper.

Elise Fritsch Lugg & Mona Gaarder: Performed the experiments; Analyzed and interpreted the data.

Ole Morten Rønning: Performed the experiments; Analyzed and interpreted the data; Contributed reagents, materials, analysis tools or data.

Birgitta Langhammer & Bente Thommessen: Analyzed and interpreted the data; Contributed reagents, materials, analysis tools or data.

### Funding statement

The work was supported by Oslo Metropolitan University -OsloMet and by UNIFORFRIMED.

### Data availability statement

Data will be made available on request.

### Declaration of interest’s statement

The authors declare no conflict of interest.

### Additional information

No additional information is available for this paper.
